# Supplementary material for: Large-scale moral machine experiment on large language models
Source: PLoS One. 2025 May 21;20(5):e0322776. doi: 10.1371/journal.pone.0322776 (PMC12094719; doi:10.1371/journal.pone.0322776)
Supplement: S1 Fig — Heatmap showing the differences between LLM and human AMCE values for nine moral preference categories. (PDF) [file pone.0322776.s001.pdf]

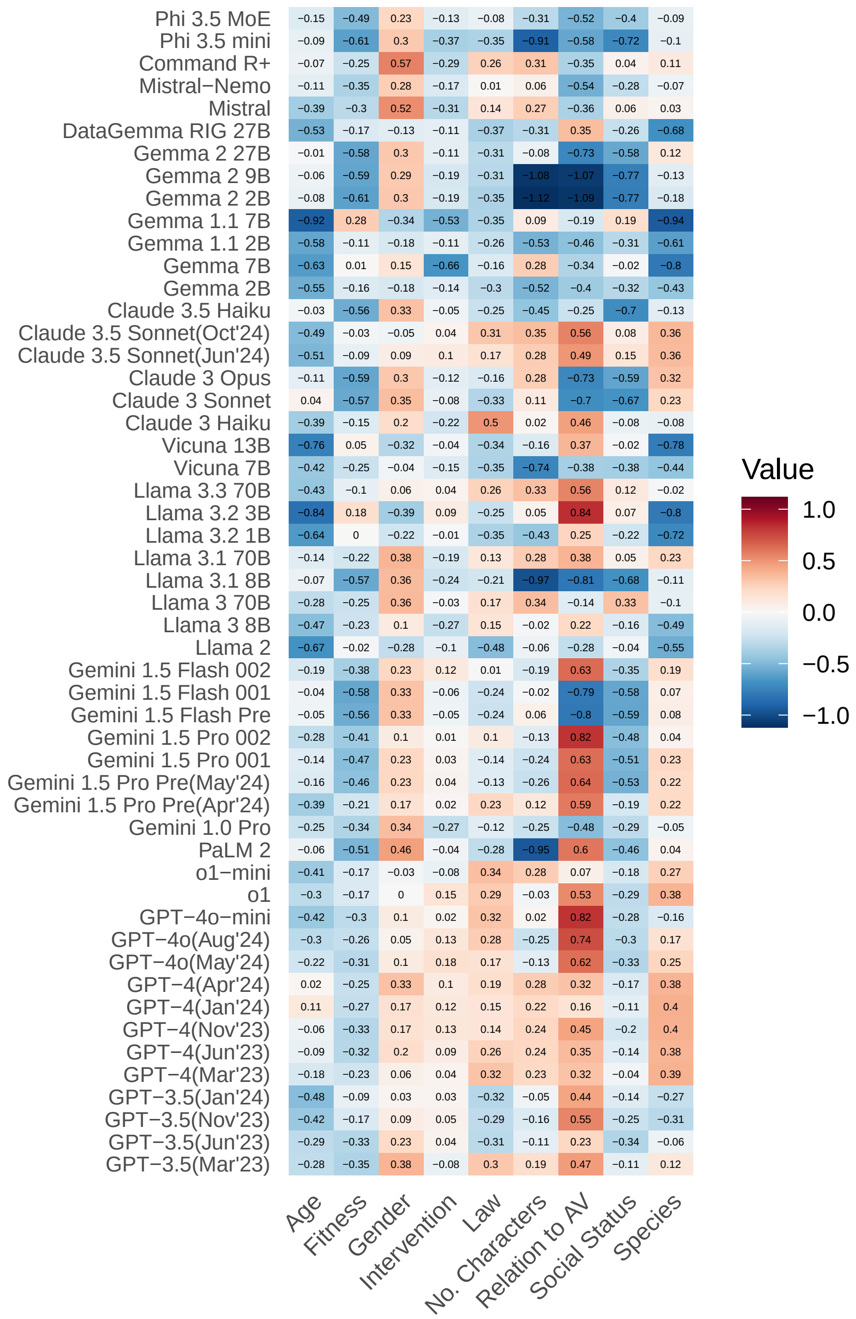
Age Fitness Gender Intervention Law No. Characters Relation to AV Social Status Species
Phi 3.5 MoE −0.15 −0.49 0.23 −0.13 −0.08 −0.31 −0.52 −0.4 −0.09
Phi 3.5 mini −0.09 −0.61 0.3 −0.37 −0.35 −0.91 −0.58 −0.72 −0.1
Command R+ −0.07 −0.25 0.57 −0.29 0.26 0.31 −0.35 0.04 0.11
Mistral−Nemo −0.11 −0.35 0.28 −0.17 0.01 0.06 −0.54 −0.28 −0.07
Mistral −0.39 −0.3 0.52 −0.31 0.14 0.27 −0.36 0.06 0.03
DataGemma RIG 27B −0.53 −0.17 −0.13 −0.11 −0.37 −0.31 0.35 −0.26 −0.68
Gemma 2 27B −0.01 −0.58 0.3 −0.11 −0.31 −0.08 −0.73 −0.58 0.12
Gemma 2 9B −0.06 −0.59 0.29 −0.19 −0.31 −1.08 −1.07 −0.77 −0.13
Gemma 2 2B −0.08 −0.61 0.3 −0.19 −0.35 −1.12 −1.09 −0.77 −0.18
Gemma 1.1 7B −0.92 0.28 −0.34 −0.53 −0.35 0.09 −0.19 0.19 −0.94
Gemma 1.1 2B −0.58 −0.11 −0.18 −0.11 −0.26 −0.53 −0.46 −0.31 −0.61
Gemma 7B −0.63 0.01 0.15 −0.66 −0.16 0.28 −0.34 −0.02 −0.8
Gemma 2B −0.55 −0.16 −0.18 −0.14 −0.3 −0.52 −0.4 −0.32 −0.43
Claude 3.5 Haiku −0.03 −0.56 0.33 −0.05 −0.25 −0.45 −0.25 −0.7 −0.13
Claude 3.5 Sonnet(Oct'24) −0.49 −0.03 −0.05 0.04 0.31 0.35 0.56 0.08 0.36
Claude 3.5 Sonnet(Jun'24) −0.51 −0.09 0.09 0.1 0.17 0.28 0.49 0.15 0.36
Claude 3 Opus −0.11 −0.59 0.3 −0.12 −0.16 0.28 −0.73 −0.59 0.32
Claude 3 Sonnet 0.04 −0.57 0.35 −0.08 −0.33 0.11 −0.7 −0.67 0.23
Claude 3 Haiku −0.39 −0.15 0.2 −0.22 0.5 0.02 0.46 −0.08 −0.08
Vicuna 13B −0.76 0.05 −0.32 −0.04 −0.34 −0.16 0.37 −0.02 −0.78
Vicuna 7B −0.42 −0.25 −0.04 −0.15 −0.35 −0.74 −0.38 −0.38 −0.44
Llama 3.3 70B −0.43 −0.1 0.06 0.04 0.26 0.33 0.56 0.12 −0.02
Llama 3.2 3B −0.84 0.18 −0.39 0.09 −0.25 0.05 0.84 0.07 −0.8
Llama 3.2 1B −0.64 0 −0.22 −0.01 −0.35 −0.43 0.25 −0.22 −0.72
Llama 3.1 70B −0.14 −0.22 0.38 −0.19 0.13 0.28 0.38 0.05 0.23
Llama 3.1 8B −0.07 −0.57 0.36 −0.24 −0.21 −0.97 −0.81 −0.68 −0.11
Llama 3 70B −0.28 −0.25 0.36 −0.03 0.17 0.34 −0.14 0.33 −0.1
Llama 3 8B −0.47 −0.23 0.1 −0.27 0.15 −0.02 0.22 −0.16 −0.49
Llama 2 −0.67 −0.02 −0.28 −0.1 −0.48 −0.06 −0.28 −0.04 −0.55
Gemini 1.5 Flash 002 −0.19 −0.38 0.23 0.12 0.01 −0.19 0.63 −0.35 0.19
Gemini 1.5 Flash 001 −0.04 −0.58 0.33 −0.06 −0.24 −0.02 −0.79 −0.58 0.07
Gemini 1.5 Flash Pre −0.05 −0.56 0.33 −0.05 −0.24 0.06 −0.8 −0.59 0.08
Gemini 1.5 Pro 002 −0.28 −0.41 0.1 0.01 0.1 −0.13 0.82 −0.48 0.04
Gemini 1.5 Pro 001 −0.14 −0.47 0.23 0.03 −0.14 −0.24 0.63 −0.51 0.23
Gemini 1.5 Pro Pre(May'24) −0.16 −0.46 0.23 0.04 −0.13 −0.26 0.64 −0.53 0.22
Gemini 1.5 Pro Pre(Apr'24) −0.39 −0.21 0.17 0.02 0.23 0.12 0.59 −0.19 0.22
Gemini 1.0 Pro −0.25 −0.34 0.34 −0.27 −0.12 −0.25 −0.48 −0.29 −0.05
PaLM 2 −0.06 −0.51 0.46 −0.04 −0.28 −0.95 0.6 −0.46 0.04
o1−mini −0.41 −0.17 −0.03 −0.08 0.34 0.28 0.07 −0.18 0.27
o1 −0.3 −0.17 0 0.15 0.29 −0.03 0.53 −0.29 0.38
GPT−4o−mini −0.42 −0.3 0.1 0.02 0.32 0.02 0.82 −0.28 −0.16
GPT−4o(Aug'24) −0.3 −0.26 0.05 0.13 0.28 −0.25 0.74 −0.3 0.17
GPT−4o(May'24) −0.22 −0.31 0.1 0.18 0.17 −0.13 0.62 −0.33 0.25
GPT−4(Apr'24) 0.02 −0.25 0.33 0.1 0.19 0.28 0.32 −0.17 0.38
GPT−4(Jan'24) 0.11 −0.27 0.17 0.12 0.15 0.22 0.16 −0.11 0.4
GPT−4(Nov'23) −0.06 −0.33 0.17 0.13 0.14 0.24 0.45 −0.2 0.4
GPT−4(Jun'23) −0.09 −0.32 0.2 0.09 0.26 0.24 0.35 −0.14 0.38
GPT−4(Mar'23) −0.18 −0.23 0.06 0.04 0.32 0.23 0.32 −0.04 0.39
GPT−3.5(Jan'24) −0.48 −0.09 0.03 0.03 −0.32 −0.05 0.44 −0.14 −0.27
GPT−3.5(Nov'23) −0.42 −0.17 0.09 0.05 −0.29 −0.16 0.55 −0.25 −0.31
GPT−3.5(Jun'23) −0.29 −0.33 0.23 0.04 −0.31 −0.11 0.23 −0.34 −0.06
GPT−3.5(Mar'23) −0.28 −0.35 0.38 −0.08 0.3 0.19 0.47 −0.11 0.12
Value
1.0
0.5
0.0
−0.5
−1.0
